# Metabolic Syndrome Is Associated with Greater Symptom Burden and Comorbidity in Chronic Obstructive Pulmonary Disease: A Secondary Analysis of the Serbian COPD Registry

**DOI:** 10.3390/medsci14020253

**Published:** 2026-05-14

**Authors:** Marija Vukoja, Marko Bojovic, Ivan Kopitovic, Sanja Dimic Janjic, Ljiljana Novkovic, Ivan Cekerevac, Ivana Stankovic, Borislav Bozanic, Sanja Hromis, Biljana Zvezdin, Zorica Lazic, Vojislav Cupurdija

**Affiliations:** 1 Faculty of Medicine, University of Novi Sad, 21000 Novi Sad, Serbia; marko.bojovic@mf.uns.ac.rs (M.B.); ivan.kopitovic@mf.uns.ac.rs (I.K.); sanja.hromis@mf.uns.ac.rs (S.H.); biljana.zvezdin@mf.uns.acrs (B.Z.); 2 The Institute for Pulmonary Diseases of Vojvodina, 21204 Sremska Kamenica, Serbia; 3 Clinic for Radiation Oncology, Oncology Institute of Vojvodina, 21204 Sremska Kamenica, Serbia; 4 Clinic for Pulmonology, University Clinical Center of Serbia, 11000 Belgrade, Serbia; sanjadimicjanjic@gmail.com; 5 Faculty of Medicine, University of Belgrade, 11000 Belgrade, Serbia; 6 Pulmonology Clinic, University Clinical Centre Kragujevac, 34000 Kragujevac, Serbia; ljiljanan1@hotmail.com (L.N.); icekerevac@gmail.com (I.C.); zoricalazickg@gmail.com (Z.L.); vojacup@gmail.com (V.C.); 7 Faculty of Medical Sciences, University of Kragujevac, 34000 Kragujevac, Serbia; 8 Clinic for Lung Diseases, University Clinical Centre of Nis, 18000 Nis, Serbia; staivana@gmail.com (I.S.); borislavbozanic@gmail.com (B.B.); 9 Faculty of Medicine, University of Nis, 18000 Nis, Serbia

**Keywords:** cardiovascular diseases, chronic obstructive pulmonary disease, comorbidity, metabolic syndrome

## Abstract

**Background:** Chronic obstructive pulmonary disease (COPD) and cardiovascular diseases (CVDs) are closely linked through shared inflammatory and metabolic pathways. Metabolic syndrome (MetS), shared by both conditions, may represent a key mechanistic link between systemic inflammation, cardiovascular disease, and COPD outcomes. **Objective:** To assess the prevalence and clinical correlates of MetS and examine whether increasing MetS burden is linked to more severe symptoms and a higher comorbidity load in patients with COPD. **Methods:** We analyzed cross-sectional data from a multicenter COPD registry. MetS was defined by the presence of at least three components: obesity, hypertension, hyperglycemia, or dyslipidemia based on clinical and medication data. Associations between MetS burden and COPD characteristics were evaluated using multivariable models adjusted for age, sex, smoking history, exacerbation status, lung function, inhaled therapy, and study center. **Results:** Among 5030 patients, MetS was present in 10.4% and was more frequent in women (11.9% vs. 9.6%, *p* = 0.01). Patients with MetS had greater symptom burden, and more frequent signs of cyanosis, cor pulmonale, and heart failure. MetS was most common in Global Initiative for Chronic Obstructive Lung Disease stage II–III and associated with higher forced expiratory volume in one second but lower forced vital capacity, with similar exacerbation rates between the groups. Cardiovascular, sleep, renal, and connective tissue comorbidities were more prevalent in patients with MetS. A dose–response relationship was observed, with each additional metabolic syndrome component independently associated with increased odds of respiratory symptoms and cardiometabolic comorbidities (all *p* < 0.01). **Conclusions:** Our findings suggest that metabolic syndrome is present in approximately 10% of patients with COPD and is associated with greater symptom burden and a higher prevalence of cardiovascular, renal, and sleep-related comorbidities. The observed stepwise relationship supports the presence of a clinically relevant cardiometabolic profile in COPD. However, given the cross-sectional registry-based design, causal inferences cannot be made, and prospective studies are needed to confirm these associations and evaluate targeted interventions.

## 1. Introduction

Chronic obstructive pulmonary disease (COPD) is one of the most prevalent chronic pulmonary disorders and remains a leading cause of morbidity and mortality worldwide [1]. Increasing evidence highlights a close and bidirectional relationship between COPD and cardiovascular disease (CVD). Longitudinal studies show that acute exacerbations of COPD are consistently associated with an elevated risk of major cardiovascular events, with this increased vulnerability persisting for up to a year after the exacerbation episode. Conversely, underlying CVD has been shown to accelerate COPD progression, contributing to higher hospitalization rates, impaired functional status, and greater mortality [2,3].

Metabolic syndrome (MetS), characterized by the coexistence of at least three components, including obesity, elevated blood pressure, hyperglycemia, and dyslipidemia, is a well-recognized risk factor for cardiovascular disease (CVD). Epidemiological data consistently demonstrate that MetS doubles the risk of cardiovascular events and increases all-cause mortality by approximately 50% [4].

Importantly, MetS is also highly prevalent among patients with COPD. A recent meta-analysis reported a pooled prevalence of Mets in COPD of 37%, with considerable heterogeneity, and higher rates in males than females [5]. Both conditions are common in individuals aged over 60 years and are associated with reduced physical activity, potentially contributing to progressive physiological decline [6]. In COPD patients, MetS is more prevalent in overweight and obese patients compared with BMI-matched healthy individuals [7].

Systemic inflammation represents a major mechanism underlying the development of chronic diseases, and both COPD and metabolic syndrome share the hallmark of chronic low-grade systemic inflammation [8]. In COPD, inflammation is driven by epithelial cell activation and chemokine spillover into the circulation, while in MetS it originates from adipokine release and pro-inflammatory cytokine secretion by visceral adipose tissue [9,10]. When these conditions coexist, they may synergistically amplify systemic inflammation, thereby heightening cardiovascular risk.

In patients with COPD, a study examining comorbidity clusters identified a distinct metabolic cluster characterized by a higher cardiovascular risk profile, reduced static hyperinflation, and lower use of inhaled therapies [11]. Consistent with this, large cohort studies have demonstrated that COPD patients with MetS experience higher rates of cardiovascular events and increased mortality [12,13]. In addition, a large retrospective cohort study demonstrated that metabolic abnormalities, irrespective of overweight or obesity status, are associated with an increased risk of unplanned readmission in patients with COPD [14].

Yet, despite its clear clinical relevance, the role of metabolic syndrome in COPD has not been fully defined as a distinct disease phenotype. One important limitation of existing studies is that comprehensive metabolic profiling is rarely available, resulting in relatively small and selected study populations. In this context, large registry-based analyses, better reflect real-world clinical practice and may provide a more accurate estimate of the true impact of metabolic syndrome in COPD.

The objective of this study was to identify and describe a MetS-associated COPD phenotype within a large multicenter registry, by determining its prevalence and examining its relationship with disease severity, symptom burden, and comorbidity patterns. In addition, we explored whether increasing numbers of MetS components were associated with a graded worsening of COPD-related clinical outcomes and health status. We hypothesized that the coexistence of MetS and COPD represents a distinct clinical phenotype marked by greater symptom burden, more advanced disease, and a higher frequency of cardiovascular comorbidities. Moreover, we hypothesized that the likelihood of adverse clinical features increases progressively with the accumulation of MetS components, consistent with a dose–response relationship.

## 2. Materials and Methods

This was a secondary analysis of the Serbian registry of patients with COPD. The details on the Registry have been previously published [15]. The study included COPD patients entered in the Registry at first visit, from 1 July 2016 to 1 October 2020. Participants were included from secondary and tertiary care settings, with data entered into the registry upon provision of written informed consent. The Registry’s design and implementation were approved by the Ethics Committee of the University Clinical Center Kragujevac and by the ethics committees of participating hospitals. All procedures were conducted in line with the ethical standards of the Declaration of Helsinki.

COPD was identified based on the presence of respiratory symptoms (including dyspnea, chronic cough, or sputum production), a history of relevant exposures such as tobacco smoking, biomass fuel exposure, or occupational inhalants, and confirmation of persistent airflow limitation on spirometry [16]. Collected variables were grouped into demographic, clinical, physiological, metabolic, comorbidity, and treatment-related categories. Clinical data included respiratory symptoms, exacerbation frequency, and lifestyle factors, particularly smoking status. Exacerbations in the previous year were classified as moderate (requiring antibiotics and/or systemic corticosteroids) or severe (requiring hospitalization in addition to treatment).

Physiological measures included spirometric indices, specifically post-bronchodilator forced expiratory volume in one second (FEV_1_) and forced vital capacity (FVC), as well as the degree of airflow limitation. Patients were further categorized into A–E groups according to the GOLD 2026 classification, based on exacerbation history and symptom burden [16].

Metabolic syndrome was defined by the presence of at least three of the following criteria: obesity (body mass index (BMI) ≥ 30 kg/m^2^), elevated blood pressure or antihypertensive treatment, hyperglycemia or use of antidiabetic therapy, and dyslipidemia or lipid-lowering treatment [17].

Comorbidities were recorded based on documented diagnoses in medical records, and the Charlson Comorbidity Index was calculated [18].

Treatment variables included respiratory medications (defined as inhaled therapies used for longer than one month) and concomitant therapies administered for the same duration. All data were obtained from the registry using standardized case report forms completed at the time of enrollment. Due to limited availability, additional assessments such as echocardiography, chest computed tomography, and the 6 min walk test were not consistently analyzed. Patients were enrolled across 21 cities; however, the majority were recruited from nine centers, including tertiary academic hospitals and secondary care settings, and treatment center was included as an adjustment variable in all multivariable models.

### Statistical Analysis

Continuous variables were summarized as mean, standard deviation, or median with interquartile range (IQR) based on data distribution. Categorical variables were expressed as frequencies and percentages. For normally distributed variables, group comparisons were performed using the *t*-test, one-way analysis of variance, or the Kruskal–Wallis non-parametric test as appropriate. Categorical variables were compared using the Chi-square test, or, when assumptions were not met, Fisher’s exact test. Multivariable logistic regression analyses were performed to identify independent associations between MetS burden and respiratory symptoms, clinical signs, and comorbidities. The models were adjusted for relevant covariates, including age, sex, smoking exposure (pack-years), exacerbations, lung function (FEV_1_% predicted and FVC% predicted), type of inhaled therapy, and treatment center. Statistical analyses were performed in JASP (version 0.95.4; University of Amsterdam, Amsterdam, The Netherlands). A *p*-value of <0.05 was considered statistically significant.

## 3. Results

The study included 5084 patients, of whom 54 had missing BMI data; therefore, 5030 patients were included in the final analysis. Baseline characteristics of the study population are shown in Table 1.

Most participants were current or former smokers, with a median smoking history of 34.3 (SD ± 31.1) pack-years. The majority of patients belonged to spirometric GOLD stages II and III. In the previous year, 2965 (59%) reported at least one moderate and 1865 (37%) at least one severe exacerbation. The frequent-exacerbator phenotype (having ≥2 moderate or ≥1 severe exacerbation) was identified in 2563 (51%) of the study population, while 3268 (65%) had at least one moderate-to-severe exacerbation in the previous year. The most common comorbidities were hypertension, followed by obesity, diabetes, and hyperlipidemia.

MetS was present in 10.4% of participants. COPD patients with MetS were more frequently female (218/1843; 11.8% vs. 302/3166; 9.5%, *p* = 0.01) and exhibited a higher overall symptom burden across all respiratory symptoms, including chronic cough, chronic sputum production, purulent sputum expectoration, dyspnea at rest and on exertion, and fatigue (Table 2). Total CAT scores (18.1 ± 5.8 vs. 15.3 ± 8.0, <0.001), as well as scores for each individual CAT component, were significantly higher in this group. Clinically, patients with MetS more often presented with clinical signs of cyanosis, cor pulmonale, and congestive heart failure, and they were more likely to require long-term oxygen therapy (LTOT).

In terms of spirometry, MetS was mostly present in patients with COPD stage II and III. COPD patients with MetS demonstrated higher FEV_1_ and lower FVC values compared to those without MetS. No significant differences were observed between groups in the number of moderate or severe exacerbations.

Comorbidity profiles revealed that COPD patients with MetS had higher rates of cardiovascular disease, obstructive sleep apnea (OSA), insomnia, connective tissue disorders, and moderate-to-severe renal disease, as well as higher Charlson comorbidity index scores.

In additional analyses, obesity was associated with a higher prevalence of metabolic syndrome compared with non-obese patients (35.1% vs. 3.04%, *p* < 0.001). Among respiratory symptoms, only chronic cough was more frequent in obese compared with non-obese patients (49.83% vs. 44.01%, *p* < 0.001). In contrast, obesity was associated with a higher prevalence of several comorbidities, including obstructive sleep apnea (3.38% vs. 0.41%, *p* < 0.001), history of myocardial infarction (6.93% vs. 5.21%, *p* = 0.03), heart failure (16.29% vs. 10.47%, *p* < 0.001), peripheral vascular disease (10.92% vs. 7.17%, *p* < 0.001), coronary artery disease (13.08% vs. 9.47%, *p* < 0.001), and depression (6.84% vs. 5.20%, *p* = 0.047), compared with non-obese patients.

**Figure 1:**
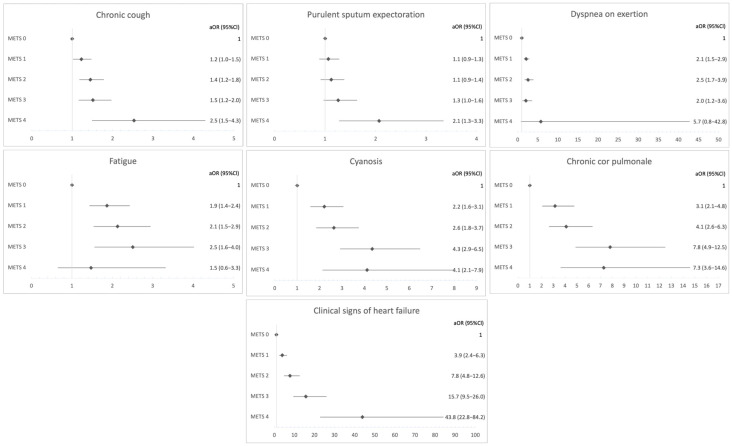
Adjusted Odds Ratios for COPD Signs and Symptoms by METS Category. Adjusted odds ratios (aORs) and 95% confidence intervals (CIs) were estimated using multivariable logistic regression models. Analyses were adjusted for age, sex, smoking exposure, exacerbation status, lung function (FEV_1_% predicted and FVC% predicted), inhaled therapy, and treatment center. MetS 0 was used as the reference category.

**Figure 2:**
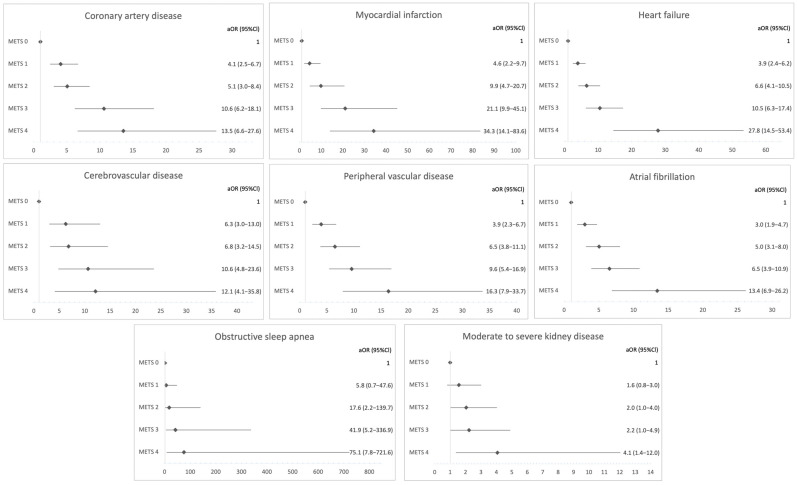
Adjusted Odds Ratios for Chronic Diseases by METS Category. Adjusted odds ratios (aORs) and 95% confidence intervals (CIs) were estimated using multivariable logistic regression models. Analyses were adjusted for age, sex, smoking exposure, exacerbation status, lung function (FEV_1_% predicted and FVC% predicted), inhaled therapy, and treatment center. MetS 0 was used as the reference category.

A stepwise, dose–response increase in symptoms (Figure 1) and comorbidities (Figure 2) was observed with each additional MetS component. Each unit increase in the number of MetS components was associated with a higher risk of chronic cough (odds ratio—OR 1.4, 95%CI 1.3–1.5, *p* < 0.001; adjusted OR—aOR 1.2, 95%CI 1.1–1.3, *p* < 0.001), chronic sputum production (OR 1.3, 95%CI 1.2–1.4, *p* < 0.001; aOR 1.1, 95%CI 1.0–1.2, *p* = 0.002), purulent sputum expectoration (OR 1.2, 95%CI 1.2–1.3, *p* < 0.001; aOR 1.1, 95%CI 1.0–1.2, *p* = 0.005), dyspnea on exertion (OR 2.9, 95%CI 2.6–3.3, *p* < 0.001; aOR 1.4, 95%CI 1.2–1.7, *p* < 0.001), fatigue (OR 2.3, 95%CI 2.1–2.6, *p* < 0.001; aOR 1.3, 95%CI 1.2–1.5, *p* < 0.001), cyanosis (OR 1.5, 95%CI 1.4–1.7, *p* < 0.001; aOR 1.4, 95%CI 1.3–1.6, *p* < 0.001), chronic cor pulmonale (OR 1.7, 95%CI 1.6–1.9, *p* < 0.001; aOR 1.6, 95%CI 1.5–1.8, *p* < 0.001) and clinical signs of heart failure (OR 2.3, 95%CI 2.1–2.5, *p* < 0.001; aOR 2.2, 95%CI 2.0–2.5, *p* < 0.001), independent of age, sex, smoking history (pack-years), exacerbation status, lung function, type of inhaled therapy, and study center.

Similar dose–response was observed with regard to comorbidities: coronary artery disease (OR 1.7, 95%CI 1.6–1.9, *p* < 0.001; aOR 1.7, 95%CI 1.5–1.9, *p* < 0.001), myocardial infarction (OR 2.2, 95%CI 2.0–2.5, *p* < 0.001; aOR 2.2, 95%CI 1.9–2.5, *p* < 0.001), heart failure (OR 2.1, 95%CI 1.9–2.2, *p* < 0.001; aOR 1.9, 95%CI 1.7–2.1, *p* < 0.001), cerebrovascular disease (OR 1.6, 95%CI 1.4–1.8, *p* < 0.001; aOR 1.4, 95%CI 1.2–1.7, *p* < 0.001), peripheral arterial disease (OR 1.9, 95%CI 1.7–2.1, *p* < 0.001; aOR 1.8, 95%CI 1.6–2.0, *p* < 0.001), atrial fibrillation (OR 1.9, 95%CI 1.7–2.1, *p* < 0.001; aOR 1.7, 95%CI 1.5–1.9, *p* < 0.001), OSA (OR 2.7, 95%CI 2.1–3.4, *p* < 0.001; aOR 2.6, 95%CI 1.9–3.6, *p* < 0.001), insomnia (OR 1.4, 95%CI 1.3–1.6, *p* < 0.001; aOR 1.3, 95%CI 1.1–1.5, *p* = 0.001), CTD (OR 1.4, 95%CI 1.1–1.7, *p* = 0.001; aOR 1.1, 95%CI 0.9–1.4, *p* = 0.28) and chronic kidney disease (OR 1.5, 95%CI 1.3–1.8, *p* < 0.001; aOR 1.3, 95%CI 1.1–1.6, *p* = 0.005).

## 4. Discussion

In this secondary analysis of a large multicenter COPD registry, the prevalence of MetS was 10%. The presence of MetS was associated with female sex, a more symptomatic disease phenotype, and a greater burden of comorbidities. After adjustment for age, sex, exacerbation status, lung function, type of inhaled therapy, and study center, MetS remained independently associated—in a dose–response manner—with an increased likelihood of respiratory symptoms, clinical signs of right heart failure, CVD, obstructive sleep apnea, insomnia, and chronic kidney disease.

MetS affects approximately 10–44% of patients with COPD, depending on the population studied, and the diagnostic criteria applied [5,12]. Despite its high prevalence, the interplay between MetS and COPD remains insufficiently explored. Both conditions share overlapping pathophysiological mechanisms, including systemic inflammation, oxidative stress, endothelial dysfunction and physical inactivity which may promote cardiovascular complications and impair functional capacity [19,20]. In line with these mechanisms, our findings show that COPD patients with MetS exhibit both a higher symptom burden and an increased prevalence of CVD. Notably, the distribution of MetS followed a U-shaped pattern, with the highest prevalence in spirometric stages II and III and fewer cases in stages I and IV. This suggests that even patients with moderate airflow limitation can experience marked symptom burden, likely driven by cardiometabolic comorbidities that exacerbate dyspnea and fatigue beyond the degree of obstruction. Consequently, reliance on obstruction-based indices such as FEV_1_ alone may underestimate disease severity in this subgroup. The lower prevalence of MetS in very severe COPD likely reflects survival bias and the weight loss with reduced BMI characteristic of advanced disease [6,11]. The lack of detailed nutritional and body composition data limited our ability to directly assess the impact of malnutrition on metabolic risk, particularly in patients with advanced COPD. The pattern of relatively preserved FEV_1_ but reduced FVC in patients with MetS further suggests a restrictive ventilatory component, possibly due to obesity, reduced chest wall compliance, or cardiometabolic effects on lung volumes. This phenotype resembles the “preserved ratio impaired spirometry” (PRISm) pattern, which is frequently associated with obesity and increased cardiovascular risk [21,22]. However, the lack of detailed body composition data precluded more precise differentiation between lean and obesity-related COPD phenotypes.

Our findings strengthen the well-established association between MetS and cardiovascular disease, while for the first time demonstrating a clear dose–response relationship within the COPD population. COPD has long been recognized as a systemic inflammatory disorder in which activation of airway epithelial cells and alveolar macrophages leads to the release of pro-inflammatory mediators including IL-6, TNF-α, CRP, and various chemokines that spillover into the systemic circulation and contribute to extrapulmonary manifestations [20,23]. Likewise, in MetS, visceral adipose tissue serves as a metabolically active organ that secretes adipokines (such as leptin and resistin) and pro-inflammatory cytokines (including TNF-α, IL-6, and MCP-1), perpetuating chronic low-grade inflammation [24]. The coexistence of COPD and MetS may intensify systemic inflammation, thereby increasing the risk of cardiovascular events, metabolic complications, and mortality [8,22]. The overlap of COPD and MetS may thus represent a distinct, high-risk phenotype characterized by heightened systemic inflammation, a greater burden of cardiovascular comorbidities, and poorer clinical outcomes. Indeed, two large longitudinal studies have demonstrated that MetS increases the incidence of cardiovascular events and overall mortality [12,13]. In a large UK primary care cohort, Karsanji et al. reported that MetS was associated with higher 5-year mortality; however, this relationship was largely attenuated after adjustment for COPD severity and coexisting comorbidities. However, individual components such as hypertension and diabetes were strong predictors of death, whereas obesity was paradoxically linked to lower mortality [12]. These findings suggest that targeting specific metabolic risk factors rather than the composite MetS definition may be more clinically relevant in COPD management. In line with these findings, our results suggest that obesity alone has a more limited association with symptom burden and comorbidities compared with the broader cardiometabolic profile captured by metabolic syndrome.

The lack of association with exacerbation rates may suggest that systemic metabolic and cardiovascular mechanisms contribute more substantially to symptom burden than airway inflammation or exacerbation susceptibility. It should be noted that our cohort was largely drawn from secondary and tertiary care, where a substantial proportion of patients were exacerbators, which may limit the generalizability of these findings to this specific population.

In addition, we observed a clear stepwise association between increasing MetS burden and the prevalence of OSA, accompanied by a higher frequency of clinical signs such as cyanosis and right heart failure. The presence of MetS in COPD patients appears to elevate the risk of developing OSA, likely through shared mechanisms involving obesity, increased upper airway collapsibility, and systemic inflammation [25,26]. The coexistence of OSA and COPD, commonly referred to as the overlap syndrome, further amplifies physiological stress, leading to more severe nocturnal hypoxemia and hypercapnia, and a higher prevalence of pulmonary hypertension and right heart failure [27]. This compounded hypoxic and inflammatory burden contributes to worse cardiovascular outcomes and increased mortality compared with either condition alone [28]. Therefore, MetS represents an important mediator linking CVD and OSA, significantly influencing the prognosis of patients with COPD.

The observed dose–response relationship between the presence of MetS and both symptom burden and cardiovascular disease highlights the potential value of implementing integrated management strategies early in the course of COPD. Targeted interventions aimed at reducing metabolic load, such as weight reduction, glycemic control, and dietary modification, should complement standard COPD pharmacotherapy and established non-pharmacological measures, such as smoking-cessation, pulmonary rehabilitation and vaccination. These findings also raise the question of whether routine cardiovascular screening should be considered in this specific phenotype to facilitate early detection and intervention. Moreover, the potential impact of anti-inflammatory treatment in these patients warrants further investigation, as two large randomized controlled trials have demonstrated that triple therapy reduces overall mortality—an effect primarily driven by a significant decrease in cardiovascular deaths among patients treated with inhaled corticosteroid–based regimens [29,30]. Our results also highlight emerging therapeutic opportunities. Recent retrospective analyses in COPD patients receiving single-inhaler triple therapy and with coexisting diabetes mellitus suggest that glucagon-like peptide-1 (GLP-1) receptor agonists may improve lung function and reduce exacerbations and mortality [31]. In addition, dual glucose-dependent insulinotropic polypeptide (GIP)/GLP-1 receptor agonists have recently been approved for the treatment of OSA in patients with obesity, a condition more prevalent in COPD patients with MetS [32]. However, the effects of these therapies on COPD-specific outcomes, particularly in patients with coexisting cardiovascular or metabolic diseases, remain insufficiently explored and require confirmation in prospective randomized controlled trials.

This study has several limitations that should be acknowledged. First, its cross-sectional design precludes establishing causal relationships between MetS, symptom burden, and comorbidities. Second, the cohort was primarily composed of patients managed in secondary and tertiary care centers, many of whom were frequent exacerbators, which may limit the generalizability of the findings to the broader COPD population. Third, the registry design did not include systematic collection of laboratory biomarkers and detailed physiological data. Consequently, MetS was defined based on available clinical diagnoses and medication use rather than standardized prospective measurements, which may have led to an underestimation of its prevalence due to missing laboratory information and a tendency to identify primarily patients with overt metabolic abnormalities. Nevertheless, this pragmatic approach is commonly used in large registry-based studies where comprehensive metabolic data are not routinely available and reflects real-world clinical practice [33,34]. Fourth, the inclusion of data collected over an extended time period represents a limitation, as temporal changes in clinical practice, diagnostic criteria, and management strategies may have influenced the observed associations. The absence of longitudinal follow-up precluded evaluation of long-term outcomes such as cardiovascular events, mortality, or progression of metabolic status. Fifth, a notable limitation of the present study is the absence of liver-specific data, particularly in the context of metabolic dysfunction-associated steatotic liver disease (MASLD), which is increasingly recognized as part of the broader spectrum of systemic metabolic dysfunction [35,36]. Although hepatic involvement is not included in the formal diagnostic criteria for MetS, substantial overlap exists in underlying mechanisms, including insulin resistance, chronic low-grade inflammation, and ectopic fat deposition. MASLD is highly prevalent in the general population and frequently coexists with COPD, with emerging evidence suggesting a shared “liver–lung axis” driven by systemic inflammation and metabolic dysregulation [37]. Moreover, MASLD in COPD has been associated with worse clinical outcomes, including increased cardiovascular risk and mortality, and may further amplify systemic disease burden. In this context, Viglino et al. reported that COPD patients with liver fibrosis had an approximately threefold higher risk of major cardiovascular events or death over 5 years compared with those without fibrosis. Importantly, neither simple steatosis nor steatohepatitis in the absence of fibrosis was associated with adverse outcomes, highlighting the prognostic relevance of liver fibrosis [38]. Given that patients with MASLD exhibit a higher prevalence of multimorbidity across cardiometabolic and renal domains, its potential contribution to the phenotype observed in our cohort is biologically plausible [39]. However, the lack of biochemical, imaging, or fibrosis-related liver parameters in our registry precluded evaluation of this important comorbidity. Future studies incorporating systematic hepatic assessment are warranted to better define the role of MASLD within the spectrum of metabolic dysfunction in COPD and to further refine phenotypic characterization. As a secondary analysis of registry data, our study is subject to inherent constraints, including limited availability of variables and the inability to capture all potentially relevant clinical and biological factors. In particular, the absence of inflammatory and insulin-related biomarkers limits mechanistic interpretation; however, these parameters are not required for the clinical diagnosis of MetS and should be considered in future studies. Additionally, the lack of data on physical activity and exercise habits represents a limitation, as these factors may influence both metabolic risk and COPD-related outcomes. The absence of detailed assessments of muscle mass and function further precluded evaluation of sarcopenia and its potential contribution to metabolic and clinical outcomes. Finally, despite adjustment for major confounders, residual confounding cannot be excluded. These limitations should be considered when interpreting the findings, as they preclude definitive conclusions.

This study has several important strengths. Although previous studies have reported an association between COPD and MetS, our study provides several novel contributions that extend beyond this established link. First, it is based on a large, multicenter, real-world cohort of 5030 patients, enhancing both the robustness and generalizability of the findings. Second, a key strength of this study is the demonstration of clear and consistent dose–response relationship between the number of MetS components and both symptom burden and comorbidity load, supporting a graded and biologically plausible effect rather than a simple binary association. Third, our results identify a clinically meaningful COPD phenotype characterized by increased symptom burden and a higher prevalence of systemic comorbidities, highlighting the relevance of cardiometabolic factors in shaping disease expression. Notably, OSA was independently associated with metabolic syndrome in our cohort, with a clear dose–response relationship across increasing MetS burden, supporting the concept of an interrelated cardiometabolic–respiratory disease cluster. These findings underscore the importance of routine OSA assessment in COPD patients, particularly in those with multiple metabolic abnormalities. Together, these findings provide a more nuanced understanding of the interplay between COPD and metabolic dysfunction and underscore the potential value of integrated cardiometabolic assessment in routine COPD care. Finally, standardized data collection within a national registry ensured methodological consistency and minimized bias.

## 5. Conclusions

In this large multicenter real-world cohort, metabolic syndrome was identified in approximately one in ten patients with COPD and was associated with greater symptom burden and a higher prevalence of cardiovascular, renal, and sleep-related comorbidities. The consistent dose–response relationship between the number of metabolic syndrome components and adverse clinical characteristics supports the presence of a clinically meaningful cardiometabolic COPD phenotype. However, these findings should be interpreted in light of several limitations, including the cross-sectional design, the use of pragmatic registry-based definitions, and the absence of laboratory-based metabolic measures, which preclude definitive causal conclusions. Nevertheless, our results suggest that incorporating cardiometabolic risk assessment into routine COPD care may help identify high-risk patients who could benefit from closer monitoring and integrated management. Future prospective and mechanistic studies are needed to confirm these associations and determine whether targeted metabolic interventions can modify disease trajectory.

## Figures and Tables

**Table 1:** Clinical characteristics of patients from the COPD Registry.

Characteristics	Values
Age yrs., mean (SD)	67.1 (9.3)
Male gender, n (%)	3166 (63.2)
Smoking status n (%)	
Never smoker	468 (9.5)
Passive smoker	65 (1.3)
Ex-smoker	2236 (45.5)
Current smoker	2146 (43.7)
Smoking pack/years, mean (SD)	34.3 (31.1)
FEV1 (% predicted), mean (SD)	52.3 (18.8)
FVC (% predicted), mean (SD)	74.6 (21.6)
FEV1/FVC (%), mean (SD)	49.5 (19.7)
GOLD spirometric stages, n (%)	
I	330 (7.0)
II	2204 (46.7)
III	1618 (34.2)
IV	570 (12.1)
Number of moderate exacerbations, mean (SD)	1.1 (1.3)
Number of severe exacerbations, mean (SD)	0.5 (0.8)
CAT, mean (SD)	15.6 (7.8)
COPD stages, n (%)	
A	865 (18.1)
B	1286 (26.9)
E	2622 (54.9)
BMI, mean (SD)	26.4 (5.4)
Hypertension, n (%)	3351 (66.6)
Coronary artery disease, n (%)	518 (10.3)
History of myocardial infarction, n (%)	282 (5.6)
Heart failure, n (%)	594 (11.8)
Atrial fibrillation, n (%)	468 (9.3)
Cerebrovascular disease, n (%)	164 (3.2)
Peripheral vascular disease, n (%)	404 (8.0)
Diabetes, n (%)	716 (14.2)
Hyperlipidemia, n (%)	727 (14.5)
Obesity, n (%)	1154 (22.9)
MetS, n (%)	523 (10.4)
MetS score, n (%)	
0	1302 (25.8)
1	2117 (42.0)
2	1088 (21.6)
3	437 (8.7)
4	86 (1.7)
Charlson comorbidity index, mean (SD)	1.9 (1.4)
Inhaled therapy	
LAMA or LABA, n (%)	1181 (27.6)
ICS monotherapy, n (%)	29 (0.7)
LABA/LAMA, n (%)	714 (16.7)
LABA/ICS, n (%)	577 (13.5)
LABA/LAMA/ICS, n (%)	1779 (41.5)

Abbreviations: SD—standard deviation, FEV_1_—forced expiratory volume in one second, FVC—forced vital capacity, GOLD—Global Initiative for Chronic Obstructive Lung Disease, CAT—COPD assessment test, BMI—body mass index, MetS—metabolic syndrome, LAMA—long-acting muscarinic antagonist, LABA—long-acting β_2_-agonist, ICS—inhaled corticosteroid.

**Table 2:** Comparison of COPD patients by MetS status.

		MetS N = 523	No MetS N = 4507	*p*
Age yrs., mean (SD)		67.5 (9.3)	67.0 (9.3)	0.28 ^a^
Male gender, n (%)		302 (9.5)	2864 (90.5)	0.01 ^b^
Smoking pack/years, mean (SD)		37.6 (30.9)	33.9 (31.1)	0.01 ^a^
Clinical presentation				
Chronic cough, n (%)		288 (55.1)	1993 (44.2)	<0.001 ^b^
Chronic sputum production, n (%)		172 (32.9)	1207 (26.8)	0.003 ^b^
Purulent sputum expectoration, n (%)		203 (38.8)	1354 (30.0)	<0.001 ^b^
Dyspnea on exertion, n (%)		502 (96.0)	3703 (82.2)	<0.001 ^b^
Dyspnea at rest, n (%)		156 (29.8)	938 (20.8)	<0.001 ^b^
Fatigue, n (%)		488 (93.3)	3619 (80.3)	<0.001 ^b^
CAT, mean (SD)		18.1 (5.8)	15.3 (8.0)	<0.001 ^a^
Cyanosis, n (%)		123 (23.8)	573 (13.0)	<0.001 ^b^
Clinical signs of cor pulmonale, n (%)		123 (23.8)	447 (10.6)	<0.001 ^b^
Clinical signs of left ventricular failure, n (%)		182 (35.1)	467 (10.6)	<0.001 ^b^
LTOT, n (%)		17 (3.2)	84 (1.9)	0.03 ^b^
FEV1 (% predicted), mean (SD)		54.1 (15.4)	52.1 (19.1)	0.01 ^a^
FVC (% predicted), mean (SD)		70.7 (18.1)	75.1 (21.9)	<0.001 ^a^
GOLD spirometric stages, n (%)	I	17 (3.4)	313 (7.4)	<0.001 ^b^
	II	282 (56.7)	1922 (45.5)	
	III	171 (34.4)	1447 (34.2)	
	IV	27 (5.4)	543 (12.8)	
Number of moderate exacerbations, mean (SD)		1.1 (1.6)	1.06 (1.3)	0.19 ^a^
Number of severe exacerbations, mean (SD)		0.5 (0.8)	0.5 (0.8)	0.70 ^a^
Exacerbators, n (%)		338 (64.6)	2930 (65.0)	0.86 ^b^
COPD stages, n (%)	A	33 (6.4)	832 (19.6)	<0.001 ^b^
	B	163 (31.6)	1123 (26.4)	
	E	320 (62.0)	2302 (54.1)	
Comorbidities				
Coronary artery disease, n (%)		114 (21.8)	404 (9.0)	<0.001 ^b^
History of myocardial infarction, n (%)		91 (17.4)	191 (4.2)	<0.001 ^b^
Heart failure, n (%)		143 (27.3)	451 (10.0)	<0.001 ^b^
Atrial fibrillation, n (%)		104 (19.8)	365 (8.1)	<0.001 ^b^
Cerebrovascular disease, n (%)		33 (6.3)	131 (2.9)	<0.001 ^b^
Peripheral vascular disease, n (%)		94 (18.1)	310 (6.9)	<0.001 ^b^
OSA, n (%)		22 (4.2)	33 (0.7)	<0.001 ^b^
Dementia, n (%)		5 (1.0)	50 (1.1)	0.75 ^b^
Connective tissue disease, n (%)		17 (3.2)	78 (1.7)	0.02 ^b^
Moderate or severe renal disease, n (%)		25 (4.8)	101 (2.2)	<0.001 ^b^
Osteoporosis, n (%)		18 (3.4)	117 (2.6)	0.26 ^b^
Depression, n (%)		33 (6.3)	292 (6.5)	0.88 ^b^
Anxiety, n (%)		32 (6.1)	202 (4.5)	0.09 ^b^
Insomnia, n (%)		48 (9.2)	253 (5.6)	0.001 ^b^
Charlson comorbidity index, mean (SD)		2.9 (1.5)	1.8 (1.3)	<0.001 ^a^
Inhaled therapy, n (%)	LAMA or LABA	157 (31.3)	1024 (27.1)	0.09 ^b^
	ICS monotherapy	1 (0.2)	28 (0.74)	
	LABA/LAMA	70 (14.0)	644 (17.0)	
	LABA/ICS	62 (12.4)	515 (13.6)	
	LABA/LAMA/ICS	211 (42.1)	1568 (41.5)	

Abbreviations: SD—standard deviation, FEV_1_—forced expiratory volume in one second, FVC—forced vital capacity, GOLD—Global Initiative for Chronic Obstructive Lung Disease, CAT—COPD assessment test, OSA—obstructive sleep apnea, MetS—metabolic syndrome, LAMA—long-acting muscarinic antagonist, LABA—long-acting β_2_-agonist, ICS—inhaled corticosteroid; statistic test: ^a^ Independent Samples *t*-Test, ^b^ Chi-square (Χ^2^) test.

## Data Availability

The datasets presented in this article are not readily available because they are part of an ongoing project and subject to privacy restrictions. Requests to access the datasets should be directed to the Zorica Lazic zoricalazickg@gmail.com.

## References

[1] de Oca M.M., Perez-Padilla R., Celli B., Aaron S.D., Wehrmeister F.C., Amaral A.F.S., Mannino D., Zheng J., Salvi S., Obaseki D. (2025). The Global Burden of COPD: Epidemiology and Effect of Prevention Strategies. Lancet Respir. Med..

[2] Nordon C., Simons S.O., Marshall J., Müllerová H., Pollack M., Bengtsson C., Hoti F., Lobier M., Salosensaari A., Santos A.C. (2025). The Sustained Increase of Cardiovascular Risk Following COPD Exacerbations: Meta-Analyses of the EXACOS-CV Studies. ERJ Open Res..

[3] Rabe K.F., Hurst J.R., Suissa S. (2018). Cardiovascular Disease and COPD: Dangerous Liaisons. Eur. Respir. Rev..

[4] Mottillo S., Filion K.B., Genest J., Joseph L., Pilote L., Poirier P., Rinfret S., Schiffrin E.L., Eisenberg M.J. (2010). The Metabolic Syndrome and Cardiovascular Risk a Systematic Review and Meta-Analysis. J. Am. Coll. Cardiol..

[5] Alrabbaie H., Al-Wardat M., Etoom M., Beauchamp M., Goldstein R., Brooks D. (2025). The Prevalence of Metabolic Syndrome in Chronic Obstructive Pulmonary Disease: A Systematic Review and Meta-Analysis. Chronic Respir. Dis..

[6] Watz H., Waschki B., Kirsten A., Müller K.-C., Kretschmar G., Meyer T., Holz O., Magnussen H. (2009). The Metabolic Syndrome in Patients with Chronic Bronchitis and COPD: Frequency and Associated Consequences for Systemic Inflammation and Physical Inactivity. Chest.

[7] Breyer M.-K., Spruit M.A., Hanson C.K., Franssen F.M.E., Vanfleteren L.E.G.W., Groenen M.T.J., Bruijnzeel P.L.B., Wouters E.F.M., Rutten E.P.A. (2014). Prevalence of Metabolic Syndrome in COPD Patients and Its Consequences. PLoS ONE.

[8] Fabbri L.M., Rabe K.F. (2007). From COPD to Chronic Systemic Inflammatory Syndrome?. Lancet.

[9] Sinden N.J., Stockley R.A. (2010). Systemic Inflammation and Comorbidity in COPD: A Result of “overspill” of Inflammatory Mediators from the Lungs? Review of the Evidence. Thorax.

[10] Chait A., den Hartigh L.J. (2020). Adipose Tissue Distribution, Inflammation and Its Metabolic Consequences, Including Diabetes and Cardiovascular Disease. Front. Cardiovasc. Med..

[11] Vanfleteren L.E.G.W., Spruit M.A., Groenen M., Gaffron S., van Empel V.P.M., Bruijnzeel P.L.B., Rutten E.P.A., Op ’t Roodt J., Wouters E.F.M., Franssen F.M.E. (2013). Clusters of Comorbidities Based on Validated Objective Measurements and Systemic Inflammation in Patients with Chronic Obstructive Pulmonary Disease. Am. J. Respir. Crit. Care Med..

[12] Karsanji U., Evans R.A., Quint J.K., Khunti K., Lawson C.A., Petherick E., Greening N.J., Singh S.J., Richardson M., Steiner M.C. (2022). Mortality Associated with Metabolic Syndrome in People with COPD Managed in Primary Care. ERJ Open Res..

[13] Noh E., Jeong H., Cho I.-S., Chang M.-S., Yu I., Park S., Lee J.-H., Lee S.J., Lee W.-Y., Yong S.J. (2024). Risk of Cardiovascular Events Associated with Chronic Obstructive Pulmonary Disease and/or Metabolic Syndrome: A Large-Scale Nationwide Population-Based Cohort Study. Int. J. Chronic Obstr. Pulm. Dis..

[14] Tian Y., Liu L., Li Y., Fan X., Wu W., Shi Y., Jiang J., Yuan Z., Dong H., Li H. (2023). The Impact of Metabolic Overweight/Obesity Phenotypes on Unplanned Readmission Risk in Patients with COPD: A Retrospective Cohort Study. Front. Physiol..

[15] Lazic Z., Stankovic I., Milenkovic B., Zvezdin B., Hromis S., Jankovic S., Cupurdija V. (2021). Characteristics of COPD Phenotypes in Serbia. Int. J. Chronic Obstr. Pulm. Dis..

[16] GOLD (2024). Global Strategy for the Diagnosis, Management, and Prevention of COPD: 2025 Report. https://goldcopd.org/2025-gold-report.

[17] Vukoja M., Tekin A., Parada N.A., Gray J.C., Mallouhi A., Roddy T., Cartin-Ceba R., Perkins N.E., Belden K.A., Cheruku S. (2024). The Association of Asthma and Metabolic Dysfunction with Outcomes of Hospitalized Patients with COVID-19. J. Allergy Clin. Immunol. Pract..

[18] Charlson M.E., Pompei P., Ales K.L., MacKenzie C.R. (1987). A New Method of Classifying Prognostic Comorbidity in Longitudinal Studies: Development and Validation. J. Chronic Dis..

[19] Roversi S., Fabbri L.M., Sin D.D., Hawkins N.M., Agustí A. (2016). Chronic Obstructive Pulmonary Disease and Cardiac Diseases. An Urgent Need for Integrated Care. Am. J. Respir. Crit. Care Med..

[20] Barnes P.J., Celli B.R. (2009). Systemic Manifestations and Comorbidities of COPD. Eur. Respir. J..

[21] Huang J., Li W., Sun Y., Huang Z., Cong R., Yu C., Tao H. (2024). Preserved Ratio Impaired Spirometry (PRISm): A Global Epidemiological Overview, Radiographic Characteristics, Comorbid Associations, and Differentiation from Chronic Obstructive Pulmonary Disease. Int. J. Chronic Obstr. Pulm. Dis..

[22] Wan E.S., Fortis S., Regan E.A., Hokanson J., Han M.K., Casaburi R., Make B.J., Crapo J.D., DeMeo D.L., Silverman E.K. (2018). Longitudinal Phenotypes and Mortality in Preserved Ratio Impaired Spirometry in the COPDGene Study. Am. J. Respir. Crit. Care Med..

[23] Gan W.Q., Man S.F.P., Senthilselvan A., Sin D.D. (2004). Association between Chronic Obstructive Pulmonary Disease and Systemic Inflammation: A Systematic Review and a Meta-Analysis. Thorax.

[24] Wajchenberg B.L. (2000). Subcutaneous and Visceral Adipose Tissue: Their Relation to the Metabolic Syndrome. Endocr. Rev..

[25] Kent B.D., McNicholas W.T., Ryan S. (2015). Insulin Resistance, Glucose Intolerance and Diabetes Mellitus in Obstructive Sleep Apnoea. J. Thorac. Dis..

[26] Soler X., Gaio E., Powell F.L., Ramsdell J.W., Loredo J.S., Malhotra A., Ries A.L. (2015). High Prevalence of Obstructive Sleep Apnea in Patients with Moderate to Severe Chronic Obstructive Pulmonary Disease. Ann. Am. Thorac. Soc..

[27] McNicholas W.T. (2016). Chronic Obstructive Pulmonary Disease and Obstructive Sleep Apnoea-the Overlap Syndrome. J. Thorac. Dis..

[28] Marin J.M., Soriano J.B., Carrizo S.J., Boldova A., Celli B.R. (2010). Outcomes in Patients with Chronic Obstructive Pulmonary Disease and Obstructive Sleep Apnea: The Overlap Syndrome. Am. J. Respir. Crit. Care Med..

[29] Lipson D.A., Barnhart F., Brealey N., Brooks J., Criner G.J., Day N.C., Dransfield M.T., Halpin D.M.G., Han M.K., Jones C.E. (2018). Once-Daily Single-Inhaler Triple versus Dual Therapy in Patients with COPD. N. Engl. J. Med..

[30] Rabe K.F., Martinez F.J., Ferguson G.T., Wang C., Singh D., Wedzicha J.A., Trivedi R., St. Rose E., Ballal S., McLaren J. (2020). Triple Inhaled Therapy at Two Glucocorticoid Doses in Moderate-to-Very-Severe COPD. N. Engl. J. Med..

[31] See X.Y., Xanthavanij N., Lee Y.-C., Ong T.E., Wang T.H., Ahmed O., Chang Y.-C., Peng C.-Y., Chi K.-Y., Chang Y. (2025). Pulmonary Outcomes of Incretin-Based Therapies in COPD Patients Receiving Single-Inhaler Triple Therapy. ERJ Open Res..

[32] Malhotra A., Grunstein R.R., Fietze I., Weaver T.E., Redline S., Azarbarzin A., Sands S.A., Schwab R.J., Dunn J.P., Chakladar S. (2024). Tirzepatide for the Treatment of Obstructive Sleep Apnea and Obesity. N. Engl. J. Med..

[33] Schütte S., Eberhard S., Burger B., Hemmerling M., Rossol S., Stahmeyer J.T. (2023). Prevalence of metabolic syndrome: Analysis based on routine statutory health insurance data. Die Inn. Med..

[34] Trabert B., Wentzensen N., Felix A.S., Yang H.P., Sherman M.E., Brinton L.A. (2015). Metabolic Syndrome and Risk of Endometrial Cancer in the United States: A Study in the SEER-Medicare Linked Database. Cancer Epidemiol. Biomark. Prev..

[35] Eslam M., Sanyal A.J., George J., on behalf of the International Consensus Panel (2020). MAFLD: A Consensus-Driven Proposed Nomenclature for Metabolic Associated Fatty Liver Disease. Gastroenterology.

[36] Targher G., Tilg H., Byrne C.D. (2021). Non-Alcoholic Fatty Liver Disease: A Multisystem Disease Requiring a Multidisciplinary and Holistic Approach. Lancet Gastroenterol. Hepatol..

[37] Al Dailaty A., Ghanem A., Abou Daher G., Chaaban T., Chatila R. (2025). Metabolic Dysfunction-Associated Steatotic Liver Disease: An Emerging Comorbidity in COPD. Ther. Adv. Chronic Dis..

[38] Viglino D., Plazanet A., Bailly S., Benmerad M., Jullian-Desayes I., Tamisier R., Leroy V., Zarski J.-P., Maignan M., Joyeux-Faure M. (2018). Impact of Non-Alcoholic Fatty Liver Disease on Long-Term Cardiovascular Events and Death in Chronic Obstructive Pulmonary Disease. Sci. Rep..

[39] Feng Q., Izzi-Engbeaya C.N., Branch A.D., Mullish B.H., Manousou P., Woodward M. (2025). The Relationships Between MASLD, Extrahepatic Multimorbidity, and All-Cause Mortality in the UK Biobank Cohort. J. Clin. Endocrinol. Metab..

